# Body contouring abdominoplasty for augmentation enterocystoplasty with continent cutaneous urinary diversion in an obese adolescent patient: A case report

**DOI:** 10.1016/j.eucr.2022.102145

**Published:** 2022-07-04

**Authors:** Joachim N. Meuli, Nuno Grilo, Beat Roth, Pietro G. di Summa

**Affiliations:** a Department of Plastic and Hand Surgery, Lausanne University Hospital, Switzerland; b Department of Urology, Lausanne University Hospital, Switzerland

**Keywords:** Augmentation cystoplasty, Abdominoplasty, Continent cutaneous urinary diversion, Appendicovesicostomy

## Abstract

Bladder exstrophy requires staged reconstruction to achieve control over bladder function. In case of failure, bladder neck closure with augmentation enterocystoplasty and appendicovesicostomy is a good surgical option. We report the case of a girl born with bladder exstrophy who, despite multiple surgical reconstructions in childhood, developed severe stress incontinence with contracted bladder and recurrent urinary tract infections during adolescence. The patient had a large abdominal pannus and in order to realize the appendicovesicostomy, we combined this intervention with a body contouring abdominoplasty to achieve tension free closure.

## 1 Introduction

Congenital malformations within the Exstrophy–Epispadias-Complex are reconstructed in stages during childhood but sometimes fail to achieve continence. In such cases, bladder augmentation and continent cutaneous diversion can provide significant relief but require a sufficiently thin abdominal pannus for the skin to be reached. When this is not the case, such as in obese patients, combined surgical procedures are required.

## 2 Case report

A girl born with congenital bladder exstrophy underwent a classical surgical reconstruction according to Grady and Mitchell at 5 days of age followed by a modified Young-Dees procedure and left ureter reimplantation at 22 months of age. Suburethral Deflux® injections combined with urethral dilatation were performed at 5, 9 and 10 years of age. At 18 years old, the patient complained of recurrent urinary tract infections and persistent stress urinary incontinence. Video-urodynamic investigations confirmed the presence of a stress urinary incontinence with low urethral pressure, a low capacity bladder with detrusor overactivity and a grade II vesicoureteral reflux.

**Fig. 1:**
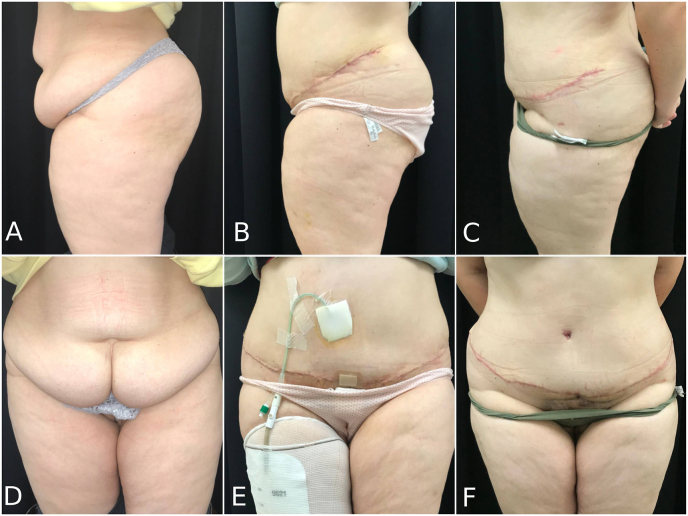
Preoperative status (A, D), 3 weeks postoperative (B, E) and 3 months postoperative (C, F).

It was decided to perform an augmentation enterocystoplasty with appendicovesicostomy and bladder neck closure. At that time, the patient presented a BMI of 35 kg/m^2^ with a significant abdominal pannus and a scarred abdominal wall with soft tissues retraction (Fig. 1). In this context, a classical appendicovesicostomy carried a high risk of insufficient appendix length to reach the umbilicus. The patient failed to lose weight over the next 3 months despite nutritional counselling. We therefore decided to perform a single stage intervention combining a body contouring abdominoplasty with the urological reconstruction.

**Fig. 2:**
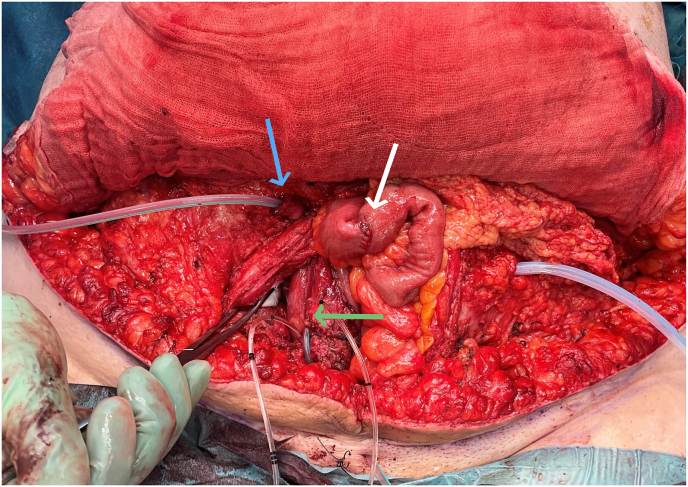
Perioperative status. Blue arrow = Mitrofanoff appendicovesicostomy. White arrow = sutured ileum. Green arrow = enterocystoplasty. (For interpretation of the references to colour in this figure legend, the reader is referred to the Web version of this article.)

We first performed the abdominoplasty following a classical technique. Once the abdominal fascia was identified, we followed this plane from caudal to cranial while avoiding excessive dissection on lateral extremities to preserve lymphatic drainage. Medially, the dissection was extended to the xiphoidal process with removal of the former neo-umbilicus. We excised the adipocutaneous excess with a safe margin to allow further correction and moved forward with the urological reconstruction. A median laparotomy incision was performed. After vesico-peritoneal dissection, the urethra was identified and resected. The bladder neck was closed after bladder preparation and ureters catheterization. The bladder was opened in a shell-like inverted U and a bladder flap was created for the tunneling of the distal part of the appendix (anti-reflux mechanism). The appendix was harvested and anastomosed to the bladder following the Mitrofanoff principle. A 25 cm segment of ileum was harvested, 25 cm proximal to the ileocecal valve. The segment was detubularized, folded in the form of a U and sutured to the remaining bladder (Fig. 2). The enterocystoplasty was then fixed in its antero-cranial part to the posterior abdominal fascia to prevent any tension on the appendix, which was catheterized with a 16 Fr. catheter.

After completion of the urological reconstruction and closure of the abdominal wall, the upper skin flap was sutured to the lower skin margin. The neo-umbilicus was created with a “V” incision and the appendix was anastomosed to the skin after defatting of the abdominal flap. The surrounding tissues were mattressed to secure the upper flap and minimize any traction on the appendicovesicostomy. Two subcutaneous drains and one intra-abdominal drain were left in place. There were no postoperative complications and the patient was discharged after 8 days. A cystography took place 3 weeks after the intervention, confirming the absence of leakage. The patient started performing self-catheterization at 6 weeks postoperative and was very satisfied with the overall result at 3 months (Fig. 1). She presented one episode of minor cutaneous stenosis of the appendicovesicostomy after 4 months that was treated conservatively with dilatation. After first dilation of the stoma and revision of catheterization technique, the patient is now able to perform self-catheterization easily and has not needed further interventions at last follow-up (12 months).

## 3 Discussion

Congenital bladder exstrophy is caused by a defect in the closure of the lower abdominal wall^1^ and is included in the wider spectrum of Exstrophy–Epispadias-Complex malformations. It occurs in approximately 1/30′000 to 1/250′000 births and is associated with cardiac anomalies. There is no consensus on the optimal surgical management strategy. Several teams using different techniques show dry continence rates ranging from 19 to 74%.^2^^,^^3^ This unfortunately means that no matter the surgical strategy used, at least 25% of the patients will require further interventions to correct low bladder volume, recurrent urinary tract infections, incontinence and/or stenosis.

The most common “last resort” surgical intervention is bladder neck closure with bladder augmentation and continent cutaneous diversion,^4^ either through an appendicovesicostomy (Mitrofanoff technique) or through a transversely tubularized bowel segment (Yang-Monti technique). This procedure allows the vast majority of patients to achieve continence^3^ and with a mean length of 81mm in adults and 68mm in children >13 years old, the appendix is sufficient in the majority of cases. In obese patients however, achieving a tension free appendicovesicostomy can be challenging. In cases that involve supplementary challenges such as abdominal scars or reduced mobility of the appendix, the available literature favors alternative techniques such as double Monti, Casale or combinations^5^ but these can be associated with poorer functional results. Continent ileocecal augmentation cystoplasty would also be a valid option for this patient, however with a higher risk of gastrointestinal complications, such as chronic diarrhea, bile acid and vitamin B12 malabsorption.

To the best of our knowledge, this report is the first describing the use of a body-contouring abdominoplasty to solve this problem by reducing the thickness and mobility of the excessive abdominal pannus. There are previously published reports of combined appendicovesicostomy and abdominoplasty but these referred to Monfort abdominoplasties in the setting of Prune Belly Syndrome, i.e. a different surgical technique for a different pathology. This technique allowed a very satisfactory functional and aesthetic result at the cost of little supplementary operating time. The patient was very satisfied with the result of the urological reconstruction as well as with the improved contouring of her abdomen. Furthermore, combining the urological procedure with the abdominoplasty improved abdominal wall stability, which itself might help the patient to improve bladder control and continence.

## Funding sources

The authors did not receive any funding for this study.
